# A retrospective analysis on the relationship between intraoperative hypothermia and postoperative ileus after laparoscopic colorectal surgery

**DOI:** 10.1371/journal.pone.0190711

**Published:** 2018-01-08

**Authors:** Ji-Won Choi, Duk-Kyung Kim, Jin-Kyoung Kim, Eun-Jee Lee, Jea-Youn Kim

**Affiliations:** Department of Anesthesiology and Pain Medicine, Samsung Medical Center, Sungkyunkwan University School of Medicine, Seoul, Republic of Korea; Charité-Universitätsmedizin Berlin, Campus Benjamin Franklin, GERMANY

## Abstract

Postoperative ileus (POI) is an important factor prolonging the length of hospital stay following colorectal surgery. We retrospectively explored whether there is a clinically relevant association between intraoperative hypothermia and POI in patients who underwent laparoscopic colorectal surgery for malignancy within the setting of an enhanced recovery after surgery (ERAS) program between April 2016 and January 2017 at our institution. In total, 637 patients were analyzed, of whom 122 (19.2%) developed clinically and radiologically diagnosed POI. Overall, 530 (83.2%) patients experienced intraoperative hypothermia. Although the mean lowest core temperature was lower in patients with POI than those without POI (35.3 ± 0.5°C *vs*. 35.5 ± 0.5°C, *P* = 0.004), the independence of intraoperative hypothermia was not confirmed based on multivariate logistic regression analysis. In addition to three variables (high age-adjusted Charlson comorbidity index score, long duration of surgery, high maximum pain score during the first 3 days postoperatively), cumulative dose of rescue opioids used during the first 3 days postoperatively was identified as an independent risk factor of POI (odds ratio = 1.027 for each 1-morphine equivalent [mg] increase, 95% confidence interval = 1.014–1.040, *P* <0.001). Patients with hypothermia showed significant delays in both progression to a soft diet and discharge from hospital. In conclusion, intraoperative hypothermia was not independently associated with POI within an ERAS pathway, in which items other than thermal measures might offset its negative impact on POI. However, as it was associated with delayed discharge from the hospital, intraoperative maintenance of normothermia is still needed.

## Introduction

A mild degree of intraoperative hypothermia, defined as a core temperature of 34.0–36°C, is an important and preventable cause of surgical complications and morbidity. This state has negative effects on bleeding and transfusion requirements, shivering, discomfort, postanesthetic recovery, morbid cardiac events, surgical wound infection, and duration of hospitalization [1]. Thus, intraoperative maintenance of normothermia is strongly recommended by several versions of the enhanced recovery after surgery (ERAS) guidelines as one of the major elements of perioperative care in colorectal surgery [2,3]. Despite the use of multiple available treatment strategies, inadvertent hypothermia is common in major colorectal surgery.

Postoperative ileus (POI) is known to be the most important factor prolonging the length of hospital stay following gastrointestinal surgery [4]. It is believed that gastrointestinal motility decreases with hypothermia, and patients with moderate to severe hypothermia (<34.0°C) may present with ileus [5]. However, no study has focused on the impact of mild intraoperative hypothermia on postoperative recovery of bowel function.

The purpose of this study was to determine whether there is a clinically relevant association between intraoperative hypothermia and POI within the setting of an ERAS pathway in adult patients who have undergone laparoscopic colorectal surgery.

## Materials and methods

The present study was registered with the Clinical Research Information Service (CRIS: https://cris.nih.go.kr/cris/en/; ref: KCT0002399) and approved by the Institutional Review Board (IRB) of the Samsung Medical Center (approval number: SMC 2017-06-036-001). All data were fully anonymized before investigators accessed them, and the requirement for informed consent was waived by the IRB.

We performed a retrospective review of consecutive patients aged 18 years or older who underwent laparoscopic colorectal surgery for malignant lesions between April 2016 and January 2017 at our institution. April 2016 was chosen as the study start date because, after that date, an ERAS pathway was fully implemented in the field of colorectal surgery at our institution. Thus, all patients were managed as per our routine ERAS pathway. The ERAS pathway included preoperative oral carbohydrate drinks, minimization of perioperative intravenous (IV) fluids and bowel preparation, early introduction of postoperative oral diet, early patient mobilization, and omission or early removal of drains, lines, nasogastric tubes, and urinary catheters.

Exclusion criteria were patients who underwent concurrent surgeries other than laparoscopic colorectal surgery, conversion to open surgery, robot-assisted laparoscopic colorectal surgery, and those on parenteral nutrition.

POI was defined as the absence of flatus and/or passage of stool or intolerance of oral intake after postoperative day 3, with radiographic confirmation of small and/or large intestinal dilatation on an abdominal radiograph [6].

### Temperature measurements and thermal management

In all cases, core temperatures were monitored continuously and recorded at 15-min intervals following the induction of anesthesia until the end of surgery using an esophageal temperature probe (Man-a-therm Esophageal, YSI 400 series; Mallinckrodt Medical, Inc., St. Louis, MO, USA). Temperature measurements at three specific time-points were obtained from the medical records: core temperature immediately after induction of anesthesia, minimum intraoperative core temperature, and temperature on arrival at the postanesthesia care unit (PACU) or intensive care unit (ICU). Postoperative temperatures were measured using infrared tympanic thermometers.

While all patients received a circulating water mattress (38°C) intraoperatively, hypothermic patients received a forced-air warming system (Bair Hugger; Augustine Medical, Eden Prairie, MN, USA) at the discretion of attending anesthesiologists.

### Anesthetic and operative management

Surgery was performed by six different surgeons, each with experience in over 200 previous laparoscopic colorectal surgeries. Laparoscopic procedures included single-incision and conventional laparoscopic colorectal surgeries. A pneumoperitoneum was established with heated and humidified carbon dioxide (CO_2_) gas using a Stryker 40L High flow Insufflator (Stryker Endoscopy, San Jose, CA, USA). Intraabdominal pressure was maintained at 12 mmHg with an insufflation rate of 20 L/min.

Anesthetic techniques were similar in all cases. Without thoracic epidural analgesia, anesthesia was induced by IV propofol or thiopental and rocuronium to provide neuromuscular blockade for endotracheal intubation. Anesthesia was maintained with total intravenous anesthesia (TIVA) using propofol and remifentanil, sevoflurane anesthesia with supplemental remifentanil infusion, or inhalation anesthesia with sevoflurane or desflurane. IV opioids (meperidine or hydromorphone) are routinely administered 30 min prior to the end of surgery for postoperative pain.

After surgery, all patients were routinely managed with IV patient-controlled analgesia (PCA) using fentanyl and/or ketorolac and rescue analgesic administration of meperidine or hydromorphone in cases of a pain numerical rating scale (NRS) score ≥4.

### Collected variables

To avoid possible spurious associations, data were collected on patient demographics, social habits such as current smoking and alcohol abuse, comorbidity status, intraoperative data (duration of surgery and anesthesia, type of surgery and anesthesia, infused volume of IV fluids, estimated blood loss), and postoperative analgesia-related data (maximum pain [NRS] score and cumulative dose of rescue opioids used during the first 3 days postoperatively). All opioid administrations were converted to IV morphine equivalent doses.

While alcohol abuse was defined as an average of 3–4 drinks per day four or more times per week, current smoking was defined as having smoked within 4 weeks of surgery and smoked at least 10 cigarettes per day for more than 1 year. Four weeks was chosen based on a meta-analysis suggestive of a reduction in surgical site infections with smoking cessation for at least 4 weeks prior to surgery [7]. Age-adjusted Charlson comorbidity index was used to assess comorbidity status. The original Charlson comorbidity index was developed in 1987, and the score is calculated by the summing the weighted scores for 19 medical conditions. Because age was determined to be a significant factor for overall survival, the age of the patients was subsequently included as a correction variable in the final Charlson index score. This modification, called the Age-Adjusted Charlson Comorbidity index, was reported to be more strongly predictive of in-hospital mortality and adverse events than other versions of the Charlson comorbidity index [8].

The type of surgery was categorized according to the portion of the bowel to be removed as right-sided colectomy (right or extended right hemicolectomy, transverse colectomy), left-sided colectomy (left or extended left hemicolectomy, anterior resection), rectal surgery (low/ultralow anterior resection, abdomino-perineal resection), and others. The length of postoperative hospital stay, days to first flatus or passage of stool, days to the first solid food intake, and the presence of wound dehiscence were also recorded.

### Statistical analysis

Statistical analyses were performed using SPSS software (ver. 18.0; SPSS Inc., Chicago, IL, USA). Univariable analyses were first performed to explore associations between each clinical characteristic and the occurrence of POI. Continuous variables were tested for normality using the Kolmogorov–Smirnov test. Non-normally and normally distributed continuous variables were analyzed with the Mann–Whitney U-test and unpaired *t*-test, respectively. Categorical variables were analyzed with the χ^2^ or Fisher’s exact test, as appropriate. To test for associations with hypothermia, temperature variables were analyzed both as continuous (*i*.*e*., in °C) and as binary categorical (*i*.*e*., hypothermic levels <36.0°C were or were not reached).

Second, a forward stepwise multivariable logistic regression analysis was conducted to determine whether hypothermia during surgery and on arrival at PACU or ICU were independent risk factors for POI. Variables with a *P* value ≤0.2 in the univariable analysis were entered into a logistic regression model. Goodness-of-fit was evaluated using the Hosmer–Lemeshow test. Independent risk factors were expressed as the odds ratio (OR) with 95% confidence interval (CI). Statistical significance was set at *P* <0.05.

## Results

Of the eligible subjects, 79 were excluded from the final analyses because of concurrent surgeries other than laparoscopic colorectal surgery (*n* = 31), robot-assisted laparoscopic colorectal surgery (*n* = 46), and conversion from laparoscopic to open surgery (*n* = 2). In total, 637 patients were analyzed, of whom 122 (19.2%) developed clinically and radiological diagnosed POI.

Core temperature immediately after anesthetic induction was similar between patients with and without POI (36.1±0.4°C *vs*. 36.1±0.4°C, *P* = 0.610). Overall, 530 (83.2%) patients experienced hypothermia at any stage intraoperatively. Of these, 378 (71.3%) had persistent hypothermia after PACU or ICU admission. The medians (interquartile ranges, [ranges]) for the lowest intraoperative core temperature were 35.3°C (35.0–35.6°C, [34.0–35.9°C]) in hypothermic patients and 36.1°C (36.0–36.3°C, [36.0–37.0°C]) in normothermic patients (*P* <0.001). When temperature was examined as a continuous variable using univariable analysis, patients with POI had a mean lowest core temperature of 0.2°C lower than those without POI (35.3±0.5°C *vs*. 35.5±0.5°C, *P* = 0.004) (Table 1).

**Table 1:** Univariable analyses of the clinical characteristics of patients with and without postoperative ileus (POI).

	POI (*n* = 122)	Non-POI (*n* = 515)	*P* value
Sex, female/male	62/60	247/268	0.570
Age (years)	63.7±12.5	61.4±11.6	0.052
BMI (kg/m^2^)	23.3±3.3	23.7±3.3	0.243
Age-adjusted Charlson comorbidity index	5.0 (3.0–6.0)	4.0 (3.0–5.0)	0.002*
Current smoking/ex- or non-smoking	15/107	85/430	0.250
Alcohol abuse, yes/no	23/99	107/408	0.635
Operator	37/22/26/22/13/2	204/74/135/61/33/8	0.093
Type of anesthesia, SEVO or DES only/ TIVA/SEVO+REMI	105/7/10	444/30/41	0.996
Type of surgery, left-sided colectomy/right-sided colectomy/rectal surgery/others	35/36/48/3	147/197/162/9	0.242
Presence of stomy, yes/no	13/109	35/480	0.146
Intravenous fluids infused (mL)	1181.7±483.3	1058.5±397.5	0.003*
Duration of anesthesia (min)	205.8±74.4	185.5±51.3	0.005*
Duration of surgery (min)	160.2±75.5	140.1±49.0	0.006*
Lowest intraoperative core temperature (°C)	35.3±0.5	35.5±0.5	0.004*
Tympanic temperature on PACU/ICU admission (°C)	35.6±0.6	35.7±0.5	0.001*
Intraoperative hypothermia, yes/no	109/13	421/94	0.044*
Hypothermia on PACU/ICU admission, yes/no	85/37	307/208	0.040*
Admission to ICU, yes/no	15/107	46/469	0.256
Maximum pain NRS score during POD 3	6.1±1.8	5.6±1.8	0.009*
Cumulative dose of rescue opioids used during POD 3 (morphine equivalents [mg])	19.5 (8.4–36.6)	13.0 (6.5–26.0)	<0.001*

BMI: body mass index; SEV: sevoflurane; DES: desflurane; TIVA: total intravenous anesthesia; REMI: remifentanil; PACU: postanesthesia care unit; ICU: intensive care unit; NRS: numerical rating scale; POD: postoperative days. Values are means ± standard deviation, medians (interquartile range), or numbers.

*Significant difference (*P* <0.05).

### Univariable and multivariable analyses: Comparison between patients with and without POI

For multivariable analyses, two temperature variables (lowest intraoperative core temperature and tympanic temperature on PACU/ICU admission) were analyzed as binary categorical variables (*i*.*e*., hypothermia or not). In addition to the temperature variables, univariable risk factors for POI were identified as high age-adjusted Charlson comorbidity index scores, large intraoperative infused volume of IV fluids, long duration of surgery and anesthesia, high maximum pain NRS score during the first 3 days postoperatively, and a large dose of rescue opioids during the first 3 days postoperatively (Table 1).

Given the high correlation between ‘duration of surgery’ and ‘duration of anesthesia’ (Pearson’s correlation coefficient = 0.981, *P* <0.001), the latter variable was removed. Thus, in addition to these seven variables, three variables associated with POI (at *P* ≤0.2) in the univariable analysis (age, presence of stomy, operator) were entered into the multivariate logistic regression analysis.

The independences of intraoperative hypothermia and hypothermia on PACU/ICU admission were not confirmed in the multivariate logistic regression analysis. However, the risk for POI was increased in a higher age-adjusted Charlson comorbidity index score and a higher maximum pain NRS score during the first 3 days postoperatively (OR = 1.310, 95% CI = 1.150–1.492, *P* <0.001; OR = 1.174, 95% CI = 1.044–1.319, *P* = 0.007, respectively). Duration of surgery and cumulative dose of rescue opioids used during the first 3 days postoperatively were also independent risk factors (OR = 1.004 for each 1-min increase, 95% CI = 1.001–1.008, *P* = 0.015; OR = 1.027 for each 1-morphine equivalent [mg] increase, 95% CI = 1.014–1.040, *P* <0.001) (Table 2).

**Table 2:** Multivariate logistic regression analysis: Independent risk factors for postoperative ileus.

Variable	Odds ratio	95% CI	*P* value
Age-adjusted Charlson comorbidity index	1.310	1.150–1.492	<0.001
Duration of surgery (min)	1.004	1.001–1.008	0.015
Maximum pain NRS score during POD 3	1.174	1.044–1.319	0.007
Cumulative dose of rescue opioids used during POD 3 (morphine equivalents [mg])	1.027	1.014–1.040	<0.001

OR: odds ratio; CI: confidence interval; NRS: numerical rating scale; POD: postoperative days.

### Relationship of intraoperative hypothermia and POI with postoperative recovery profiles

Patients presenting with POI had significantly longer times to first flatus or passage of stool and to the first solid food intake than those without POI. These differences in the recovery of bowel function resulted in a prolonged postoperative hospital stay in patients with POI compared with those without POI (Table 3).

**Table 3:** Recovery profiles of patients with and without postoperative ileus (POI), and those with and without intraoperative hypothermia.

	POI (*n* = 122)	Non-POI (*n* = 515)	*P* value
Length of postoperative hospital stay (days)	8.0 (7.0–9.0)	6.0 (5.0–7.0)	<0.001*
Time to the first solid food intake (days)	3.0 (2.0–4.3)	2.0 (2.0–3.0)	<0.001*
Time to pass flatus or passage of stool (days)	3.0 (3.0–5.0)	3.0 (2.0–3.0)	<0.001*
	Intraoperative hypothermia (*n* = 530)	Intraoperative normothermia (*n* = 107)	*P* value
Length of postoperative hospital stay (days)	6.9±2.9	6.2±1.8	0.027*
Time to the first solid food intake (days)	2.0 (2.0–4.0)	2.0 (2.0–3.0)	0.003*
Time to pass flatus or passage of stool (days)	3.0 (2.0–3.0)	3.0 (2.0–3.0)	0.075
Wound dehiscence	7	0	0.608

Values are means ± standard deviation, medians (interquartile range), or numbers.

*Significant difference (*P* <0.05).

When comparing patients with and without intraoperative hypothermia, patients with hypothermia showed significant delays in both progressing to solid food intake and discharge from the hospital compared with those without. However, there were no significant differences in terms of time to pass flatus or passage of stool. Wound dehiscence was observed only in patients with intraoperative hypothermia (*n* = 7), in which one case required a repair operation under general anesthesia (Table 3).

## Discussion

The present study showed that POI is a common phenomenon that occurs in 19.2% of adult patients undergoing laparoscopic colorectal surgery, even in the context of an ERAS pathway. Although several items of the ERAS pathway are used to prevent POI, this high rate of POI may be attributed to poorly controlled modifiable risk factors, as well as the presence of unmodifiable risk factors.

During the planning of the present study, we considered intraoperative hypothermia as a major modifiable risk factor of POI. Previous experimental [9] and clinical [10, 11] studies showed that hypothermia promotes paralytic ileus, and its detrimental effect on gastrointestinal motility appears to be temperature-dependent. Splanchnic hypoperfusion due to vasoconstriction [11] or alterations in the pacemaker current initiating contractions of the intestinal smooth muscle [9] is believed to be the primary cause of such an ileus.

However, in contrast to our expectations, intraoperative hypothermia was found not to be an independent risk factor of POI. The most likely explanation for this unexpected result is that several items of the ERAS pathway (including a laparoscopic surgical approach) induced a relatively small intraoperative temperature change and further attenuated the impact of hypothermia on the establishment of POI.

While it is evident that moderate to severe hypothermia resulted in an increased risk of ileus, it is uncertain whether a mild degree of hypothermia has a similar impact on ileus. Previous studies showing a relationship between hypothermia and ileus evaluated critical patients undergoing moderate induced hypothermia (32–34°C) [10, 12]. In contrast, all hypothermic patients in the present study fulfilled the criteria for mild hypothermia (median; 35.3°C, interquartile range; 35.0–35.6°C), which might be mainly attributed to a laparoscopic approach. Compared to an open approach, a laparoscopic approach exhibits reduced heat loss by minimizing bowel exteriorization [13]. In addition, the surgical condition of CO_2_ pneumoperitoneum may affect our results. In the present study, insufflated CO_2_ gas was warmed to 37°C and humidified with 100% relative humidity. Localized direct warming [14] and local insufflation of humidified and warm CO_2_ gas [15] during open surgery resulted in increased local oxygen delivery or perfusion. In this regard, compared to the use of unwarmed or dry CO_2_ gas, the use of warmed and humidified CO_2_ gas may additionally exert favorable local effects on gut blood flow through exposure of the bowel, thus decreasing the risk of POI.

In the present study, all patients received several items of the ERAS pathway, such as use of laparoscopy, avoidance of routine use of a drain and nasogastric tube, preoperative provision of carbohydrate-loaded liquids, restricted perioperative fluid substitution, and early postoperative feeding and mobilization. As a result, the well-defined benefits of the ERAS program (other than thermal items) can offset the potential disadvantages of intraoperative hypothermia in terms of recovery of bowel function.

In the present study, four variables (high age-adjusted Charlson comorbidity index score, long duration of surgery, high maximum pain NRS score during the first 3 days postoperatively, and a large cumulative dose of rescue opioids used during the first 3 days postoperatively) were identified as independent risk factors for POI.

The negative impact of postoperative opioid use on gastrointestinal motility and its dose dependence are well established in various surgical settings [4, 6]. Poorly controlled postoperative pain also promotes POI either directly (increased sympathetic tone) or indirectly (delayed mobilization and increased consumption of opioid analgesics). A recent retrospective study involving 11,397 patients who underwent open or laparoscopic colon resection showed that higher age-adjusted Charlson comorbidity index score was an independent predictor of prolonged POI [16].

Longer operative time is a risk factor for POI in the setting of colorectal surgery [17]. Longer operative time may indicate extended surgery, technical difficulties, and/or an increased inflammatory response, any of which may directly promote POI [4, 6]. Owing to the lack of significant variability in surgical expertise, the learning curve does not likely contribute to operative time differences in our group of surgeons.

Because there appears to be a relationship between the risk of POI and the anatomical location of the surgery, we categorized the type of surgery into four groups (right-sided colectomy, left-sided colectomy, rectal surgery, and others) and evaluated its associations with the occurrence of POI. As a result, the type of surgery was not associated with POI (*P* = 0.242). Although one retrospective study suggested that right-sided laparoscopic colectomy is associated with a four-fold higher risk of POI than a left-sided laparoscopic colectomy [18], another study failed to demonstrate a relationship between the risk of POI and the anatomical location of the surgery [19]. Thus, considering our results together, the impact of the anatomical location of the surgery on the risk of POI may not be substantial.

In the present study, we reconfirmed the association between intraoperative hypothermia and increased length of hospital stay, although it was not independently associated with POI. As the etiology of POI is multifactorial, several factors other than intraoperative hypothermia can affect the establishment of POI. Interestingly, wound dehiscence was observed only in patients with intraoperative hypothermia (*n* = 7), although it did not reach statistical significance. The adverse impacts of hypothermia on wound infection and healing have been well defined [1].

This study had several potential limitations. One major limitation was the use of retrospective methods for data collection. We did not have information on the doses of opioids administered via IV PCA (due to the use of disposable PCA devices), which may have contributed to the POI in addition to the dose of rescue opioids used during the first 3 days postoperatively. In cases of disposable PCAs device without a recording function of PCA demand, the amount of drug used by a patient is generally estimated by visual examination of the volume left in the device. However, this method is far from accurate [20]. Because all patients received the standardized clinical pathway for postoperative pain control (*e*.*g*., similar PCA regimens and an identical protocol for postoperative rescue analgesia), the cumulative dose of rescue opioids used during the first 3 days postoperatively may proportionally reflect the total amount of opioid used during the postoperative period.

In addition, we were unable to assess the relationship between the duration of intraoperative hypothermia and the occurrence of POI. If intraoperative hypothermia affects the development of POI, the length and severity of hypothermia are important considerations. The relatively long recording interval (15 min) of intraoperative temperatures made it difficult to quantify the exact duration of intraoperative hypothermia.

In conclusion, intraoperative hypothermia was a common problem affecting up to 83.2% of laparoscopic colorectal surgical patients, but it was not independently associated with POI. As a laparoscopic surgical approach usually leads to a mild degree of hypothermia, the negative impact of intraoperative hypothermia on POI can be offset by other factors in the ERAS pathway. However, when considering the association of intraoperative hypothermia with delayed discharge from the hospital along with the well-defined benefits of maintaining normothermia, the importance of maintaining intraoperative normothermia should not be ignored.

## Supporting information

S1 File Original dataset. dx.doi.org/10.17504/protocols.io.mdyc27w.
(XLSX)

S2 File Age-adjusted Charlson comorbidity index. dx.doi.org/10.17504/protocols.io.mbzc2p6.
(DOCX)
