# Supplementary material for: A retrospective analysis on the relationship between intraoperative hypothermia and postoperative ileus after laparoscopic colorectal surgery
Source: PLoS One. 2018 Jan 8;13(1):e0190711. doi: 10.1371/journal.pone.0190711 (PMC5757986; doi:10.1371/journal.pone.0190711)
Supplement: S2 File — dx.doi.org/10.17504/protocols.io.mbzc2p6. (DOCX) [file pone.0190711.s002.docx]

**Table. Age-adjusted Charlson comorbidity index**

| Score | Condition |
| --- | --- |
| 1 | Myocardial infarction (history, not ECG changes only)  Congestive heart failure  Peripheral disease (includes aortic aneurysm ≥ 6 cm)  Cerebrovascular disease: CVA with mild or no residua or TIA  Dementia  Chronic pulmonary disease  Connective tissue disease  Peptic ulcer disease  Mild liver disease (without portal hypertension, includes chronic hepatitis)  Diabetes without end-organ damage (excludes diet-controlled alone) |
| 2 | Hemiplegia  Moderate or severe renal disease  Diabetes with end-organ damage  Tumor without metastasis (exclude if > 5 yr from diagnosis)  Leukemia (acute or chronic)  Lymphoma |
| 3 | Moderate or severe liver disease |
| 6 | Metastatic solid tumor  AIDS (not just HIV positive) |
| For each decade > 40 years of age, a score of 1 is added to the above score. | |

ECG: electrocardiography; CVA: cerebrovascular accident; TIA: transient ischemic attack, AIDS: acquired immunodeficiency syndrome; HIV: human immunodeficiency virus.
